# Associations between the neighbourhood environment characteristics and physical activity in older adults with specific types of chronic conditions: the ALECS cross-sectional study

**DOI:** 10.1186/s12966-016-0377-7

**Published:** 2016-04-21

**Authors:** Anthony Barnett, Ester Cerin, Casper J. P. Zhang, Cindy H. P. Sit, Janice M. Johnston, Martin M. C. Cheung, Ruby S. Y. Lee

**Affiliations:** 1 Institute of Health and Ageing, Australian Catholic University, Melbourne, VIC Australia; 2 School of Public Health, The University of Hong Kong, Hong Kong, China; 3 Department of Sports Science and Physical Education, Faculty of Education, The Chinese University of Hong Kong, Hong Kong, China; 4 Elderly Health Service, Department of Health, The Government of Hong Kong Special Administrative Region, Hong Kong, China

**Keywords:** Active transport, Walking, Recreation, Mobility, Chronic disease, Ultra-dense cities

## Abstract

**Background:**

Neighbourhood characteristics may influence physical activity (PA), which has positive effects on the health of older adults. Older adults with chronic conditions are less active and possibly more affected by environmental factors than their peers. Understanding neighbourhood characteristics associated with PA specific to older adults with chronic conditions is currently lacking. This cross-sectional study aimed to assess the associations between the neighbourhood environment and various forms of PA in older adults with and without visual impairment, hearing impairment, musculoskeletal disease and/or genitourinary disease.

**Methods:**

Neighbourhood environment and PA data were collected in Hong Kong older adults (*N* = 909) from 124 preselected neighbourhoods stratified for walkability and socioeconomic status. Generalized linear models and zero-inflated negative binomial models with robust standard errors were used to examine associations of perceived neighbourhood environment characteristics, and the moderating effects of having specific chronic conditions, with PA outcomes.

**Results:**

Thirteen perceived neighbourhood characteristics were associated with older adults’ PA in the expected direction irrespective of their health condition. Nine neighbourhood characteristics had associations with PA that were dependent on hearing impairment, vision impairment, musculoskeletal disease or genitourinary disease. In general, they were stronger in participants with than without a specific chronic condition.

**Conclusions:**

Maximizing the potential for PA in older adults who have lower levels of physical functionality due to chronic conditions may require neighbourhood characteristics specific to these groups.

## Background

The importance of engagement in physical activity (PA) for older adults’ health is well documented. For example, in older adults, PA has positive effects on grey matter volume [1], quality of life, and autonomous living [2], while it is negatively associated with mortality [3], cognitive decline [4], frailty [5], sarcopenia [6], and cardiovascular disease risk [7].

Neighbourhood characteristics facilitating engagement in PA are likely to have a large-scale, population-level, long-term effect on PA. Identification of these characteristics can inform urban planning interventions and ensure that these interventions address the needs of low-active groups, such as older adults with certain chronic conditions. Both perceived and objectively measured characteristics of the neighbourhood built environment, such as access to services and safety, have been found to be associated with PA in older adults [8, 9]. However, an understanding of neighbourhood characteristics associated with PA specific to older adults with chronic conditions, which could inform urban design that encourages PA in these populations who may have lower levels of physical functionality, is currently lacking.

Due to chronic condition-related mobility limitations, the effects of the neighbourhood environment on PA in older adults with specific chronic conditions may differ from those seen in the general older adult population and may vary across different types of chronic conditions. While comparisons in other countries are lacking, individuals in the USA with visual impairment, musculoskeletal disease, hearing impairment or incontinence have been found to engage in fewer steps per day and/or lower levels of PA than their peers without that condition [10–13]. All four of these conditions have also been associated with higher levels of fear of falling [14–16]. Neighbourhood environment characteristics associated with lower level of confidence in being active outdoors in older adults with fear of falling include walking on slippery or uneven surfaces or in a crowded place [14]. In comparison to those without visual impairment, visually impaired adults have mobility limitations [17], and are less likely to travel from home [18], both of which can restrict participation in PA [19, 20]. Neighbourhood characteristics such as poor access to services and public transport, crowdedness, high traffic volume, hilly and slippery streets, and poor street lighting may negatively impact to a greater extent on PA engagement in this population than in non-visually impaired healthy older adults. With regard to musculoskeletal diseases, PA has positive disease-specific effects on osteoarthritis (OA) of the knee; reducing pain, improving physical performance and reducing depressive symptoms [11]. However, compared with those without, people with osteoarthritis are more likely to have problems with walking [21]. Access to various services and public transport, presence of sitting facilities and absence of steep slopes are possible neighbourhood attributes that may encourage PA in older adults with arthritis. For older adults with genitourinary conditions, easy access to services and toilets may also encourage PA. Hearing impairment has been associated with greater odds of poorer function in activities of daily living and general PA, memory limitation and confusion [22]. In addition to influences of some of the previously mentioned neighbourhood environmental characteristics, attributes associated with noise and other external threats (e.g., crowdedness, traffic and crime) may have greater effect on PA in those with this condition compared to those with no hearing impairment.

The prevalence rates of visual impairment, musculoskeletal diseases, genitourinary diseases, and hearing impairment in ≥65-year-olds are substantially higher than in younger age groups [23–25], with musculoskeletal problems such as osteoarthritis and joint pain reaching ~50 % prevalence in many aging populations [26]. Thus, to promote aging in place, it is important to identify aspects of the neighbourhood environment that would facilitate the maintenance of health-enhancing levels of PA and independent community living in these chronic-condition subgroups. This paper specifically looked at the associations between the perceived neighbourhood environment and various forms of PA in Hong Kong older adults; and whether these associations differed between those with and without diagnosed vision, hearing, musculoskeletal and genital/urinary related conditions.

## Methods

Data for this paper were from the Active Lifestyle and the Environment in Chinese Seniors (ALECS) study, an observational, primarily cross-sectional study [27]. This study received ethical approval by the University of Hong Kong Human Research Ethics Committee for Non-Clinical Faculties (EA270211) and the Department of Health (Hong Kong SAR).

### Participants

The study sample were 909 Hong Kong older adults (mean (SD) age, 76 (6) years; gender, 34 % male) living in 124 preselected neighbourhoods (Tertiary Planning Units – TPUs; smallest administrative units with census-level data) stratified by walkability and socio-economic status. This stratification maximizes the range of neighbourhood built environment variables related to transport-related walkability while controlling for neighbourhood-level socio-economic status based on median household income [28]. The walkability index was computed from Census, and Geographic Information Systems data [29], as a function of net residential density, land use mix and intersection density [30]. Details about the selection of TPUs are given elsewhere [27].

As the Hong Kong Personal Data (Privacy) Ordinance imposes restriction on data owners in making available residential address and other contact data of potentially eligible participants [31], recruitment was undertaken in person from Elderly Health Centres of the Department of Health, Hong Kong Special Administrative Region (72 % of the sample) and elderly community centres (28 % of the sample) in close proximity to the selected TPUs. Participant recruitment eligibility criteria were: a) being 65 years of age or older; b) being Cantonese speaking and able to communicate verbally; c) having lived in one of the selected TPUs for at least six months; d) being able to walk unassisted for at least 10 m; and e) being cognitively intact (Mini-Mental State Examination score > 22) [32].

Upon recruitment, verification of eligibility, and provision of written informed consent to take part in in the study, each participant undertook a one-to-one interview with a trained research assistant to collect information on socio-demographics, health-related problems, perceived neighbourhood characteristics, and participation in PA. All surveys were interviewer-administered. A modest incentive (HK$50) and participation certificate were provided and participants also entered a draw to win one of three gift certificates valued at HK$2,000. The overall response rate was 71 % of the 1280 eligible potential participants. The number of non-eligible participants (because they had serious health problems and could not walk or because they lived outside the target, selected areas) were 322. That is, in total, we contacted 1602 participants. The sample socio-demographic and relevant health status characteristics are presented in Table 1.

**Table 1:** Descriptive statistics for sociodemographic characteristics (*N* = 909)

Sociodemographic characteristics	Value
Educational attainment (%)	
No formal education	21
Primary school	36
Secondary school	30
Post-secondary	13
Household with car (%)	28
Type of housing (%)	
Public and aided	43
Private (purchased)	51
Renting	6
Living alone (%)	23
Area of residence (%)	
High walkable, high socioeconomic status	25
High walkable, low socioeconomic status	28
Low walkable, high socioeconomic status	25
Low walkable, low socioeconomic status	22
Diagnosed chronic condition prevalence (%)	
None of the 4 chronic conditions	24
Vision impairment	58
Cataracts	56
Glaucoma	6
Musculoskeletal	49
Polyarthritis	44
Osteoporosis	10
Adhesive capsulitis of shoulder	3
Genitourinary	22
Prostrate	14
Incontinence	8
Hearing impairment	18
Two or more of the 4 chronic conditions	48
Number of other medical conditions, Mean (SD), range	1.78 (1.39) 0–7
Self-reported visual/auditory clarity (total sample)	
See things clearly with or without glasses	
Very clearly	65
Clearly	21
Not very clearly	12
Not clearly at all	1
Hear clearly with or without audiophones	
Very clearly	81
Clearly	11
Not very clearly	7
Not clearly at all	1
Physical activity variables, Mean (SD), median (IQR)	
Non-walking moderate-to-vigorous (weekly minutes)	337 (493), 210 (420)
Within neighbourhood walking for transport (weekly minutes)	169 (205), 120 (180)
Within neighbourhood walking for transport (weekly frequency)	8 (8), 7 (11)
Within neighbourhood walking for recreation (weekly minutes)	137 (220), 0 (210)
Within neighbourhood walking for recreation (weekly frequency)	3 (4), 0 (7)

*SD* standard deviation, *IQR* interquartile range

### Measures

#### Exposures

Perceived neighbourhood environmental characteristics postulated to be related to walking were assessed using the Neighbourhood Environment Walkability Scale for Chinese Seniors (NEWS-CS) [33], a valid and reliable questionnaire adapted for Chinese elders and based on the Neighbourhood Environment Walkability Scale [34]. For the purpose of this study, we used subscales assessing perceived access to destinations, features related to accessibility and pedestrian infrastructure, density, personal safety, traffic safety, aesthetics, and availability of sitting facilities (see Table 2 for details).

**Table 2:** Perceived neighbourhood environment attributes - NEWS-CS (*N* = 909)

Variable	Mean	SD	Min	Max
Access of destinations				
Land use mix – diversity	4.3	0.8	1	4.9
Land use mix - access to services	3.7	0.6	1	4
Proximity of public transport	4.4	0.9	1	5
Proximity of recreational facilities	3.0	0.9	1	5
Features related to accessibility and pedestrian infrastructure				
Street connectivity	3.5	0.6	1	4
Pedestrian infrastructure	3.6	0.5	1	4
Indoor places for walking	2.6	1.1	1	4
Physical barriers to walking	1.6	0.6	1	3.7
Easy access of residential entrance	3.7	0.7	1	4
Bridge/overpass connecting services	1.8	1.1	1	4
Density				
Dwelling density	651	152	263	1000
Crowdedness	1.6	0.8	1	4
Personal safety				
Presence of people	3.4	0.7	1	4
Social disorder/littering	1.7	0.7	1	4
Crime	1.4	0.7	1	4
Traffic safety				
Traffic and road hazards	1.7	0.6	1	4
Traffic speed	2.7	0.6	1	4
Fence separating sidewalk and traffic	3.0	1.2	1	4
Aesthetics	2.7	0.8	1	4
Sitting facilities	2.5	1.2	1	4

*NWQ-CS* Neighbourhood Walking Questionnaire – Chinese version for Seniors. All perceived environmental attributes except for Residential density and Land-use mix – diversity were assessed using a 4-point Likert scale. Land-use mix – diversity was assessed by the perceived walking proximity from home to a list of destinations, with responses ranging from 1 to 5 min (5) to 30 min (1) walking distance. Residential density items used a 5-point scale with ratings weighted relative to the average residential density that a specific item represents (34). The score on each NEWS-CS subscale represents the level of presence of a specific attribute. For example, a higher score on the land use mix – diversity subscale indicates a higher degree of diversity in destination types within a neighbourhood; a higher score on the crime subscale indicates higher perceived crime in the neighbourhood

#### Outcomes

Self-reported weekly minutes of moderate-to-vigorous PA other than walking were assessed using the Chinese version of the International Physical Activity Questionnaire (IPAQ-C) – Short (last 7 days). The IPAQ-C has been shown to have good reliability and validity in Chinese older adults [35]. Weekly frequency and total minutes of within-neighbourhood, transportation and recreational walking (in a usual week) were assessed via the Neighbourhood Walking Questionnaire – Chinese version for Seniors (NWQ-CS) [36], adapted from the Neighbourhood Physical Activity Questionnaire [37]. Participants reported the frequency and duration (total minutes per week) of walking for transport and recreation undertaken within their neighbourhood (an area up to 15-minute walk from home).

#### Covariates and moderators

Data on sex, age, education (up to primary education; secondary or higher education), marital status (married or cohabiting, widowed, and other), living arrangements (living alone or living with others), housing (private, public and aided, renting), and car in the household (yes or no) were collected during the face-to-face interview. The presence or absence of chronic conditions in study participants was determined using information from clinical health-problems checklists obtained from the EHCs (based on medical staff assessments) or the participant for those recruited outside the EHC, i.e., at elderly community centres. For the purpose of this paper, the specific chronic conditions examined were hearing impairment, vision impairment (glaucoma, cataracts), genitourinary (urinary incontinence, disorder of prostrate) and/or musculoskeletal (polyarthritis excluding osteoarthritis of spine, osteoporosis, adhesive capsulitis of shoulder) problems. Indicator variables were created to indicate the presence or absence of a specific category of chronic condition and the number of co-morbid conditions (e.g., cancer, psychiatric diseases, etc.) for each participant. Given that members of the EHCs are considered to be more health conscious and, hence, likely more physically active than non-members [38], a variable denoting the type of recruitment centre (elderly community centre vs. EHC) was entered as a co-variate. Indicator (dummy) variables of specific chronic conditions and type of recruitment centre were also considered as moderators of the environment-PA associations. Although not a main aim of this study, the latter variable was considered as an additional moderator of environment-PA associations to ascertain the generalizability of the findings to non-members of the EHCs.

### Data analyses

Descriptive statistics were computed for all variables. Generalized linear models (GLMs) and zero-inflated negative binomial models with robust standard errors accounting for TPU-level clustering were used to examine associations of perceived neighbourhood environment characteristics, and the moderating effects of having specific chronic conditions, with PA outcomes [39]. Weekly minutes of non-walking moderate-to-vigorous PA and frequency and weekly minutes of within-neighbourhood recreational walking were modelled using zero-inflated negative binomial regression models because they had a larger number of observations with 0 values (e.g., 0 weekly minutes reported) than expected under a negative binomial distribution. These types of models produce two sets of regression estimates: the odds ratios (OR) of reporting non-zero frequency/minutes of PA associated with a 1 unit increase in the environmental correlates; and the proportional difference in non-zero outcome values associated with a 1 unit increase in the environmental correlates for those reporting some PA/walking [40]. Frequency and weekly minutes of within-neighbourhood walking for transport were modelled using GLMs with negative binomial variance and logarithmic link function, yielding regression estimates of proportional differences in frequency/weekly minutes of walking associated with a 1 unit increase in the environmental correlates.

The first aim of this study was to estimate confounder-adjusted associations of perceived neighbourhood characteristics with various forms of PA. For this purpose, a first set of models estimated the confounder-adjusted associations of single perceived environmental correlates with PA outcomes. Curvilinearity in associations was assessed using polynomials. The moderating effects of diagnosed chronic conditions on the environment-PA associations (second study aim) were assessed by adding appropriate interaction terms to the previous regression models. Significant interactions were examined by estimating associations for participants with and without a specific type of chronic condition group (e.g., with and without visual impairments). Significant environmental correlates and interaction terms were entered in fully-adjusted multiple-environmental-variable main-effect and main-effect + interaction regression models. Only those correlates and interaction terms that showed a significant independent contribution to the PA outcome (*p* < .05) were retained in final multiple-environmental-variable models. All analyses were conducted in Stata 12.0.

## Results

Participants reported high levels of non-walking PA and within-neighbourhood walking (Table 1). Both weekly minutes of non-walking PA and within-neighbourhood walking for transport exceeded the WHO recommended minimum for total PA in older adults [41]. The participant population prevalence of chronic conditions examined in this paper was highest for vision impairment (58 %), followed by musculoskeletal (49 %), genitourinary (22 %) and hearing impairment (18 %) (Table 1). The average number (SD) of other medical conditions per participant was 1.78 (1.39). Significant differences in the odds of engaging in non-walking PA were found between participants with vision impairment and those without, with the latter being more likely to engage than the former (OR = 1.45, 95 % CI: 1.10, 1.92; *p* = .009). No other significant differences in self-reported PA were found between participants with and without the examined chronic conditions.

### Associations of Neighbourhood Environment Characteristics with Physical Activity

For the total sample, when adjusting for socio-demographics and health status, all PA outcomes were found to be significantly associated with multiple perceived neighbourhood characteristics (Table 3). Within neighbourhood walking for transport was associated with 13 of the 20 neighbourhood characteristics in the single variable models (SEV), the greatest number for the three types of PA. Associations with 7 neighbourhood characteristics were still present in the models after adjusting for other significant environmental variables (MEV). For example, 1 unit increases in access to services or traffic and road hazards were associated with a 30 % increase in weekly minutes and a 14 % decrease in weekly frequency of within-neighbourhood walking for transport, respectively. Ten and 7 attributes were significantly associated with non-walking PA in the SEV and MEV models, respectively. For those who participated in non-walking PA, strong associations with pedestrian infrastructure and crowdedness (inverse) were found, with a 1 unit increase in the environmental characteristic associated with a 25 % increase and 23 % decrease in weekly minutes, respectively. Crime (inverse) and neighbourhood aesthetics had the strongest associations with the odds of participating in non-walking PA. A 1 unit increase in these variables was associated with a 32 % decrease and 35 % increase in the odds of participating in non-walking PA. Within-neighbourhood walking for recreation was associated with 9 neighbourhood characteristics in the SEV models and 7 in the MEV models. Presence of people was the strongest predictor of being a walker for recreation with a 1 unit increase associated with close to a 40 % increase in the odds of walking. For those who did walk, 1 unit increases in physical barriers to walking and crowdedness were each associated with a 14 % decrease in weekly minutes of within-neighbourhood walking for recreation.

**Table 3:** Associations of perceived neighbourhood characteristics with physical activity outcomes – main effects

		Non-walking PA		Within-neighbourhood walking for transport		Within-neighbourhood walking for recreation			
Perceived neighbourhood characteristics	Model	Zero versus non-zero weekly minutes	Non-zero weekly minutes	Weekly frequency	Weekly minutes	Zero versus non-zero weekly frequency	Non-zero weekly frequency	No versus non-zero weekly minutes	Non-zero weekly minutes
		OR (95 % CI) *p*-value	e^b^ (95 % CI) *p*-value	e^b^ (95 % CI) *p*-value	e^b^ (95 % CI) *p*-value	OR (95 % CI) *p*-value	e^b^ (95 % CI) *p*-value	OR (95 % CI) *p*-value	e^b^ (95 % CI) *p*-value
Access of destinations									
Land use mix – diversity	SEV	1.195 (0.988, 1.445) 0.067	0.999 (0.886, 1.125) 0.983	1.219*** (1.14, 1.334) <0.001	1.118* (1.017, 1.231) 0.022	1.066 (0.878, 1.296) 0.518	1.037 (0.960, 1.119) 0.355	1.062 (0.879, 1.286) 0.214	0.999 (0.896, 1.114) 0.980
Land use mix - access to services	SEV	0.999 (0.779, 1.281) 0.995	1.150* (1.016, 1.301) 0.027	1.369*** (1.220, 1.536) <0.001	1.384*** (1.206, 1.588) <0.001	1.350* (1.033, 1.765) 0.028	1.060 (0.934, 1.204) 0.362	1.326* (1.017, 1.729) 0.037	0.991 (0.829, 1.185) 0.920
	MEV	-	-	1.159* (1.018, 1.319) 0.026	1.304*** (1.151, 1.477) <0.001	-	-	-	-
Proximity of public transport	SEV	1.070 (0.919, 1.249) 0.382	1.012 (0.940, 1.090) 0.751	0.963 (0.900, 1.030) 0.274	1.013 (0.918, 1.20) 0.785	1.043 (0.887, 1.228) 0.613	1.051* (1.001, 1.093) 0.013	1.047 (0.890, 1.132) 0.574	1.100* (1.019, 1.188) 0.015
	MEV	-	-	-	-	-	1.050* (1.000, 1.104) 0.050	-	1.118** (1.039, 1.204) 0.003
Proximity of recreational facilities	SEV	1.231* (1.019, 1.486) 0.031	1.077 (0.979, 1.185) 0.129	1.102** (1.028, 1.182) 0.006	1.091* (1.003, 1.187) 0.042	1.116 (0.963, 1.299) 0.144	0.973 (0.915, 1.035) 0.398	1.115 (0.963, 1.292) 0.145	0.962 (0.871, 1.060) 0.434
	MEV	1.249* (1.035, 1.508) 0.021	-	-	-	-	-	-	-
Accessibility & pedestrian infrastructure									
Street connectivity	SEV	1.125 (0.912, 1.383) 0.272	0.873* (0.767, 0.994) 0.041	1.250*** (1.125, 1.388) <0.001	1.174* (1.031, 1.337) 0.015	1.129 (0.895, 1.422) 0.306	0.990 (0.902, 1.087) 0.831	1.120 (0.888, 1.412) 0.338	1.070 (0.964, 1.187) 0.198
	MEV	-	-	1.104* (1.010, 1.232) 0.046	-	-	-	-	-
Pedestrian infrastructure	SEV	0.914 (0.965, 1.189) 0.501	1.190* (1.014, 1.395) 0.033	1.314*** (1.150, 1.501) <0.001	1.282*** (1.108, 1.481) 0.001	1.123 (0.884, 1.496) 0.427	1.070 (0.971, 1.178) 0.172	1.127 (0.854, 1.490) 0.398	1.029 (0.890, 1.190) 0.696
	MEV	-	1.248** (1.089, 1.431) 0.002	1.149* (1.005, 1.315) 0.043	-	-	-	-	-
Indoor places for walking	SEV	1.119 (0.949, 1.320) 0.182	0.900* (0.819, 0.988) 0.027	1.082* (1.014, 1.152) 0.017	1.106* (1.021, 1.199) 0.014	1.131 (0.972, 1.317) 0.110	1.056* (1.000, 1.117) 0.050	1.139* (1.002, 1.293) 0.046	1.031 (0.959, 1.109) 0.410
	MEV	-	-	-	-	-	1.056* (1.001, 1.113) 0.042	-	-
Physical barriers to walking	SEV	0.849 (0.647, 1.064) 0.155	1.162 (0.928, 1.455) 0.191	0.812*** (0.747, 0.883) <0.001	0.830* (0.718, 0.960) 0.012	0.983 (0.771, 1.252) 0.889	0.910* (0.840, 0.986) 0.022	0.986 (0.775, 1.255) 0.909	0.894* (0.803, 0.997) 0.044
	MEV	-	-	0.863*** (0.792, 0.941) <0.001	0.892* (0.794, 0.999) 0.049	-	0.919* (0.850, 0.995) 0.036	-	0.864* (0.770, 0.970) 0.013
Easy access of residential entrance	SEV	1.153 (0.903, 1.470) 0.253	1.109 (0.977, 1.258) 0.111	1.050 (0.595, 1.150) 0.294	1.020 (0.926, 1.123) 0.683	1.150 (0.927, 1.426) 0.204	1.036 (0.957, 1.121) 0.384	1.148 (0.929, 1.422) 0.201	0.978 (0.872, 1.096) 0.696
Bridge/overpass connecting services	SEV	1.034 (0.875, 1.223) 0.696	0.994 (0.918, 1.077) 0.880	1.034 (0.988, 1.081) 0.147	1.039 (0.976, 1.107) 0.232	1.023 (0.882, 1.186) 0.764	1.026 (0.962, 1.094) 0.442	1.028 (0.891, 1.188) 0.704	1.018 (0.949, 1.092) 0.623
Density									
Dwelling density	SEV	0.999 (0.998, 1.000) 0.151	1.000 (0.999, 1.001) 0.644	1.003** (1.001, 1.005) 0.004	1.000 (0.999, 1.001) 0.222	1.000 (0.999, 1.001)	1.000 (0.999, 1.000)	1.001 (0.999, 1.002) 0.159	1.000 (0.999, 1.000) 0.871
Crowdedness	SEV	1.146 (0.921, 1.426) 0.222	0.795*** (0.704, 0.898) <0.001	1.000 (0.918, 1.089) 0.998	0.970 (0.862, 1.092) 0.619	0.845 (0.687, 1.040) 0.111	1.065 (0.971, 1.168) 0.176	0.837* (0.701, 0.999) 0.048	0.895* (0.804, 0.997) 0.043
	MEV	-	0.773*** (0.685, 0.873) <0.001	-	-	-	-	-	0.872* (0.782, 0.972) 0.013
Personal safety									
Presence of people	SEV	1.005 (0.772, 1.309) 0.969	0.896* (0.803, 1.000) 0.049	1.257*** (1.139, 1.388) <0.001	1.215*** (1.085, 1.361) <0.001	1.441** (1.146, 1.811) 0.002	1.036 (0.961, 1.116) 0.358	1.455*** (1.164, 1.822) <0.001	1.078 (0.974, 1.191) 0.148
	MEV	-	0.885* (0.802, 0.976) 0.015	1.146* (1.032, 1.273) 0.011	1.112* (1.001, 1.235) 0.047	1.392** (1.100, 1.761)	-	1.404** (1.116, 1.765) 0.004	-
Social disorder/littering	SEV	1.259 (0.991, 1.598) 0.059	0.994 (0.876, 1.128) 0.928	0.972 (0.897, 1.053) 0.486	0.956 (0.859, 1.064) 0.410	1.094 (0.868, 1.379) 0.443	1.054 (0.976, 1.140) 0.178	1.097 (0.874, 1.377) 0.424	1.048 (0.953, 1.151) 0.334
Crime	SEV	0.718** (0.564, 0.913) 0.007	1.010 (0.982, 1.229) 0.100	0.883** (0.811, 0.960) 0.004	0.870* (0.775, 0.976) 0.017	0.794* (0.656, 0.959) 0.017	0.975 (0.906, 1.050) 0.502	0.792* (0.657, 0.956) 0.015	0.929 (0.786, 1.098) 0.388
	MEV	0.680** (0.531, 0.873) 0.002	-	-	-	-	-	-	-
Traffic safety									
Traffic and road hazards	SEV	0.950 (0.726, 1.242) 0.709	1.100 (0.930, 1.301) 0.267	0.913 (0.805, 1.036) 0.159	0.826 (0.711, 0.959) 0.012	0.806 (0.599, 1.083) 0.153	1.042 (0.900, 1.207) 0.584	0.820 (0.614, 1.094) 0.178	1.094 (0.947, 1.265) 0.219
	MEV	-	-	-	0.860 (0.762, 0.971) 0.015	-	-	-	-
Traffic speed	SEV	1.225 (0.931, 1.611) 0.147	1.112 (0.974, 1.269) 0.117	0.913 (0.821, 1.016) 0.095	0.967 (0.848, 1.102) 0.615	0.830 (0.634, 1.089) 0.178	0.952 (0.879, 1.032) 0.231	0.820 (0.651, 0.970) 0.048	0.890 (0.793, 1.021) 0.101
	MEV	-	-	-	-	-	-	-	0.911* (0.808, 0.994) 0.036
Fence separating sidewalk and traffic	SEV	0.934 (0.828, 1.053) 0.256	1.073 (0.973, 1.184) 0.155	1.025 (0.974, 1.080) 0.342	1.045 (0.976, 1.117) 0.203	0.904* (0.816, 1.000) 0.049	1.054* (1.006, 1.105) 0.029	0.913 (0.825, 1.010) 0.077	1.034 (0.972, 1.100) 0.290
Aesthetics	SEV	1.297* (1.038, 1.620) 0.022	0.849** (0.723, 0.958) 0.008	1.066 (0.956, 1.19) 0.253	1.097* (1.000, 1.204) 0.050	1.061 (0.890, 1.264) 0.512	1.007 (0.936, 1.084) 0.845	1.069 (0.903, 1.265) 0.439	0.986 (0.896, 1.085) 0.777
	MEV	1.349* (1.147, 1.883) 0.011	0.857** (0.761, 0.964) 0.010	-	-	-	-	-	-
Sitting facilities	SEV	1.072 (0.923, 1.245) 0.353	1.073* (1.003, 1.133) 0.011	1.044 (0.988, 1.104) 0.125	1.081* (1.006, 1.162) 0.033	1.166* (1.024, 1.330) 0.020	1.007 (0.952, 1.065) 0.812	1.171* (1.050, 1.331) 0.016	1.009 (0.943, 1.080) 0.794
	MEV	-	1.079** (1.020, 1.141) 0.008	-	-	1.137* (1.004, 1.288) 0.044	-	1.140* (1.018, 1.275) 0.023	-

*Notes:* All models are adjusted for socio-demographics, type of recruitment centre, specific types of chronic conditions (visual impairment, hearing impairment, genitourinary diseases, and musculoskeletal diseases), and number of other medical conditions. OR, odds ratio; e^b^, antilogarithm of regression coefficient (denoting proportional difference in outcome associated with 1 unit difference in environmental correlate); CI, confidence intervals; PA, physical activity; SEV, single-environmental-variable (only single environmental variable entered in the model); MEV, multiple-environmental-variable (multiple significant environmental variables entered in the model); −, not applicable. * *p* < .05; ** *p* < .01; *** *p* < .001

### Chronic Conditions as Moderators of Neighbourhood Environment-PA Associations

All four chronic condition groups had a number of significant neighbourhood environment-PA associations that differed from those without the chronic condition (Table 4). These differences were typically characterized by not significant associations in those without the condition and significant associations in those with the condition.

**Table 4:** Moderating effects of diagnosed chronic conditions on associations between perceived neighbourhood characteristics and physical activity outcomes

Chronic conditions	Perceived neighbourhood characteristics (PNC)	Physical activity outcome (PAO)	Diagnosed chronic condition	Associations between PNC and PAO		
				OR or e^b^	95 % CI	*p*-value
Vision impairment	Land use mix – access to services	Non-zero weekly minutes of non-walking PA	No	0.943	0.827, 1.076	0.383
			Yes	1.189*	1.028, 1.374	0.020
	Physical barriers to walking	Non-zero weekly minutes of non-walking PA	No	0.978	0.814, 1.174	0.809
			Yes	0.750***	0.633, 0.889	<0.001
	Aesthetics	Weekly frequency of within-neighbourhood walking for transport	No	1.249**	1.073, 1.452	0.004
			Yes	0.950	0.837, 1.076	0.420
		Weekly minutes of within-neighbourhood walking for transport	No	1.248***	1.092, 1.427	<0.001
			Yes	1.003	0.817, 1.232	0.977
Hearing impairment	Land use mix – diversity	Non-zero weekly minutes of non-walking PA	No	0.943	0.827, 1.076	0.545
			Yes	1.189*	1.028, 1.375	0.020
	Land use mix – access to services	Non-zero weekly frequency of within-neighbourhood walking for recreation	No	1.315***	1.155, 1.432	<0.001
			Yes	1.794***	1.288, 2.499	<0.001
		Non-zero weekly minutes of within-neighbourhood walking for recreation	No	0.946	0.790, 1.134	0.548
			Yes	1.665***	1.260, 2.200	<0.001
	Pedestrian infrastructure	Non-zero weekly minutes of non-walking PA	No	1.129	0.947, 1.346	0.176
			Yes	1.810***	1.454, 2.253	<0.001
	Crime	Non-zero weekly minutes of non-walking PA	No	1.163	0.947, 1.435	0.159
			Yes	0.866*	0.769, 0.976	0.018
	Traffic and road hazards	Non-zero weekly minutes of non-walking PA	No	1.164	0.975, 1.390	0.093
			Yes	0.715*	0.501, 0.993	0.045
Musculoskeletal disease	Land use mix – diversity	Non-zero weekly minutes of non-walking PA	No	0.999	0.735, 1.324	0.993
			Yes	1.398**	1.105, 1.770	0.005
	Proximity to recreational facilities	Zero vs. non-zero weekly minutes of non-walking PA	No	0.903	0.690, 1.181	0.456
			Yes	1.655***	1.333, 2.056	<0.001
	Easy access of residential entrance	Weekly minutes of within-neighbourhood walking for transport	No	0.911	0.792, 1.046	0.186
			Yes	1.392*	1.060, 1.830	0.018
Genitourinary disease	Land use mix – access to services	Weekly minutes of within-neighbourhood walking for transport	No	1.233**	1.067, 1.426	0.005
			Yes	2.115***	1.292, 3.482	<0.001
		Zero vs. non-zero weekly minutes of within-neighbourhood walking for recreation	No	1.150	0.853, 1.552	0.361
			Yes	2.598**	1.268, 5.323	0.009
	Pedestrian infrastructure	Weekly frequency of within-neighbourhood walking for transportation	No	1.249**	1.074, 1.452	0.004
			Yes	1.657***	1.281, 2.142	<0.001
		Weekly minutes of within-neighbourhood walking for transportation	No	1.169	0.993, 1.376	0.061
			Yes	1.807***	1.332, 2.453	<0.001
	Traffic and road hazards	Weekly minutes of within-neighbourhood walking for transportation	No	0.952	0.798, 1.135	0.583
			Yes	0.501***	0.384, 0.653	<0.001

*Notes:* All models are adjusted for socio-demographics, type of recruitment centre, specific diagnosed chronic condition type (visual impairment, hearing impairment, genitourinary diseases, and musculoskeletal diseases), number of other medical conditions, and other significant perceived neighbourhood characteristics and environment by chronic condition interaction effects. OR, odds ratio; e^b^, antilogarithm of regression coefficient (denoting proportional difference in outcome associated with 1 unit difference in environmental correlate); CI, confidence intervals; PA, physical activity; −, not applicable. * *p* < .05; ** *p* < .01; *** *p* < .001

#### Vision impairment

While there was no association found for those not visually impaired, land use mix – access to services (positive) and physical barriers to walking (inverse) were associated with non-zero weekly minutes of non-walking PA in the visually impaired. For those visually impaired, a 1 unit increase in land mix use – access to services was associated with a 19 % increase in non-walking PA and a 1 unit increase in physical barriers to walking was associated with a 25 % decrease in non-walking PA. Weekly frequency and minutes of within-neighbourhood walking for transport were associated with aesthetics in those without visual impairment; in the visually impaired there was no association.

#### Hearing impairment

Land use mix – access to services was more strongly associated with frequency of neighbourhood walking for recreation in those with than without hearing impairment. Access to services was also associated with non-zero minutes of neighbourhood walking for recreation (OR = 1.665) in the hearing impaired only. Land use mix – diversity, pedestrian infrastructure, crime (inverse) and traffic and road hazards (inverse) were all significantly associated with non-zero weekly minutes of non-walking PA in those with hearing impairment only.

### Musculoskeletal disease

For those with musculoskeletal disease who participated in non-walking PA, weekly minutes were associated with land use mix – diversity. The odds of participating in non-walking PA were associated with proximity to recreational facilities and ease of access of residential entrance was associated with weekly minutes of neighbourhood walking for transport only in those with musculoskeletal disease.

#### Genitourinary disease

Weekly minutes of within-neighbourhood walking for transport was associated with access to services, pedestrian infrastructure and traffic and road hazards (inverse) in those with genitourinary disease. For access to services, the association was also significant in those without genitourinary disease. However, associations were stronger in those with genitourinary disease. A 1 unit increase in traffic and road hazards was associated with a 50 % decrease in within-neighbourhood walking for transportation in those with genitourinary disease only. There was also a significant association between access to services and likelihood of neighbourhood walking for recreation (OR = 2.598) in those with genitourinary disease.

## Discussion

This study is, to the best of our knowledge, the first to examine perceived neighbourhood environment correlates of PA in older adults with specific chronic conditions. We estimated the associations between the perceived neighbourhood environment and self-reported non-walking MVPA, walking for transport and walking for recreation in a community sample of Hong Kong Chinese older adults and in subgroups with and without at least one of chronic vision, hearing, musculoskeletal or genitourinary conditions. Several neighbourhood characteristics were significantly or more strongly associated with PA in those with than without the studied chronic conditions. These findings suggest the need to consider specific environmental attributes when planning friendly neighbourhoods for older adults with chronic conditions and associated lower levels of physical functionality, a large proportion of the older adult population.

A substantial number of associations of PA with perceived neighbourhood attributes were not moderated by diagnosed chronic conditions, indicating these neighbourhood attributes were similarly important PA correlates across diagnostic groups. These included: land-use mix – diversity and proximity to recreational facilities in relation to within-neighbourhood walking for transport; physical barriers to walking and crime in relation to walking for transport and recreation; aesthetics in relation to non-walking PA; proximity of public transport; street connectivity; indoor places for walking; dwelling density; crowdedness; the presence of people; traffic speed; and sitting facilities.

The number of significant environmental correlates for walking for transport and walking for recreation were similar to but slightly higher than that found in a previous study of Hong Kong elders [9, 42]. The difference may be due to the larger sample size (909 vs 484) and thus increased power to detect effects and/or differences in the cohorts’ subjects. Also, selection criteria for the previous cohort included ‘no mobility problems’ as compared to ‘with and without mobility problems (but able to walk unassisted for >10 m)’ for the current cohort. The more population inclusive criteria in the current study may have resulted in a higher level of reactivity to environmental influences in participants, possibly resulting in larger statistical effects.

Contrary to findings elsewhere [10–13], with the exception of the odds of engaging in non-walking PA in the vision impaired compared to those without, no significant differences in self-reported PA were found between participants with and without the studied chronic conditions. This may be due to influences of the high-density urban environment and/or cultural factors [43], and reflect the high levels of PA in Hong Kong older adults, with 50 % of Hong Kong residents ≥ 60 years of age reporting exercising every day [44]. Also, people with specific chronic conditions may purposefully increase their PA levels based on the recommendations of their health centres or they may tend to overestimate the time spent being physically active due to increased levels of perceived effort. However, this similarity in levels of PA across groups does not negate the need to encourage PA friendly neighbourhood environments for less robust groups, such as those with chronic conditions.

### Vision

Access to services and physical barriers to walking (inverse) were significantly associated with non-zero weekly minutes of non-walking PA only in those with impaired vision. The first of these was hypothesized, as was hilly streets (inverse), a sub-item of physical barriers to walking in the NEWS-CS [33]. Our other hypothesized associations with PA (access to public transport; crowdedness (inverse); high traffic volume (inverse); street lighting, a sub-item of pedestrian infrastructure) in the visually impaired were equally important to other participants (Table 3). The combination of associations between fear of falling and visual impairment in Hong Kong elders [14] may result in a preference not to venture further from home than is essential. So, high access to services may encourage non-walking PA in nearby facilities. Also, a high percentage (86 %) of the sample, reported clear visual clarity with (if visually impaired) or without glasses. This may indicate that many of the visually impaired were not greatly limited by this impairment in daily life, possibly also explaining the small number of specific associations in this group. Lastly, aesthetics was associated with walking for transport in non-visually impaired, but not in the visually impaired. This may be expected due to the visually impaired being less able to view and appreciate landscapes and to Hong Kong green areas being very hilly and hence challenging to the visually impaired.

### Hearing

Hearing impairment in older adults has been associated with a number of conditions [22], including brain atrophy [45], which may negatively impact on levels of PA and health-related quality of life. The negative effects of hearing impairment associated memory and confusion [22] on PA are likely exacerbated by challenging and unsafe neighbourhood environments. Observed negative PA associations with crime and traffic and road hazards and positive association with pedestrian infrastructure in this group appear to support this contention. Our hypothesis that crowdedness, with its associated noise levels and possible perceived threats due to confusion, may also be negatively associated with PA in the hearing impaired was supported as it applied to the whole sample (including the hearing impaired).

### Musculoskeletal

Attaining sufficient PA had been associated with better health-related quality of life and reduced depressive symptoms in older adults with arthritis, the most prevalent type of musculoskeletal disease in the study sample (44 % of study participants - Table 1), and with reducing pain, improving physical performance and reducing depressive symptoms in those with osteoarthritis [11, 46]. As musculoskeletal pain is prevalent among older Hong Kong adults [47], and shown to be linked with mobility impairment [48], neighbourhood environment characteristics that facilitate access to destinations may be especially important in enabling the accumulation of sufficient PA in this population. Two of the three neighbourhood characteristics positively associated with PA in this group only (land use mix - diversity, reflecting distance to destinations; easy access to residential entrance) fit into this category. The third, proximity to recreational facilities, was associated with the likelihood of participating in non-walking PA in the total sample, with a stronger association in the musculoskeletal group. While land use mix –diversity was linked to PA in this group, and therefore expected to reduce depressive symptoms, it has also been positively associated with depression in older Australian males, with the presence of retail appearing to be very important [49]. The authors suggested this association may be due to the large parking lots typically associated with retail centres in Australia, which older adults may find unsafe. As car ownership is low in Hong Kong and provision of parking minimal, very few people use cars when shopping.

### Genitourinary

Women with urinary incontinence have impaired quality of life, take fewer steps per day and have poor physical performance [50]. Also, quality of life is higher in prostate cancer survivors who participate in non-vigorous physical activity and walking [51]. A neighbourhood environment conducive to PA should have a positive influence on quality of life in older adults with these problems. In this study, walking for transport was associated with access to services, pedestrian infrastructure and traffic and road hazards (inverse) in those with genitourinary conditions only. The odds of participating in walking for recreation were also associated with access to services only in those with genitourinary conditions. High levels of access to services, pedestrian infrastructure quality and minimal traffic and road hazards could be expected to minimize time spent travelling to destinations, an important consideration to many with genitourinary conditions. In Hong Kong, access to services is typically associated with access to public toilets, making it easier those with urinary incontinence to venture further from home.

## Conclusion

This study is the first to show that, in addition to characteristics that are associated with PA in older adults irrespective of their health condition, there are specific neighbourhood characteristics associated with PA in older adults with hearing impairment, vision impairment, musculoskeletal disease and genitourinary disease. The findings are important for ensuring good urban design for these populations who are likely to find their neighbourhood environment more challenging than their peers. Further research is needed in other geographical locations to explore the generalizability of these findings.

This study has several limitations. It is cross-sectional in nature, cannot account for self-selection bias or prove causality. It is possible that older adults may choose to live in neighbourhoods that have characteristics suited to their needs in relation to chronic conditions. However, this source of validity threat is less relevant in Hong Kong older adults due to low levels of car ownership and residential mobility. Participant perceived, rather than objective measurements of neighbourhood characteristics were used. Regular engagement in PA may lead to greater awareness and, therefore being more likely to report attributes of their neighbourhood environment in more detail. Self-reported measures were used to determine levels of PA. While not expected to be as accurate as objective measures (e.g., accelerometry), this allowed exploration of different PA contexts (within-neighbourhood) and types (walking versus non-walking) which are potentially influenced by different neighbourhood characteristics. Objective measurement using accelerometry only allows identification of total ambulatory activities, and matching this to location using GPS monitoring is not feasible in high-density areas such as Hong Kong where the satellite signal is often lost.
